# Research Progress on the Surface Modification of Basalt Fibers and Composites: A Review

**DOI:** 10.3390/ma18051164

**Published:** 2025-03-05

**Authors:** Miaomiao Zhu, Mingming Zhu, Ruoxin Zhai, Wuwei Zhu, Jiabei He

**Affiliations:** 1 Shaanxi Provincial Academy of Building Research Co., Ltd., Xi’an 710082, China; 18191857913@163.com (M.Z.); 18792503776@163.com (R.Z.); zhuwuweijjy@163.com (W.Z.); 2 Advanced Materials Research Center, Northwest Institute for Nonferrous Metal Research, 96 Weiyang Road, Xi’an 710016, China

**Keywords:** basalt fiber, surface modification, resin matrix, composites, interfacial bonding strength

## Abstract

Fiber-reinforced resin composites (FRRCs) are widely used in several fields such as construction, automotive, aerospace, and power. Basalt fiber (BF) has been increasingly used to replace artificial fibers such as glass fiber and carbon fiber in the production of BF-reinforced resin matrix composites (BFRRCs). This preference stems from its superior properties, including high temperature resistance, chemical stability, ease of manufacturing, cost-effectiveness, non-toxicity, and its natural, environmentally friendly characteristics. However, the chemical inertness of BF endows it with poor compatibility, adhesion, and dispersion in a resin matrix, leading to poor adhesion and a weak BF–resin interface. The interfacial bonding strength between BF and resin is an important parameter that determines the service performance of BFRRC. Therefore, the interfacial bonding strength between them can be improved through fiber modification, resin–matrix modification, mixed enhancers, etc., which consequently upgrade the mechanical properties, thermodynamic properties, and durability of BFRRC. In this review, first, the production process and properties of BFs are presented. Second, the mechanical properties, thermodynamic properties, and durability of BFRRC are introduced. Third, the modification effect of the non-destructive surface-modification technology of BF on BFRRC is presented herein. Finally, based on the current research status, the future research direction of BFRRC is proposed, including the development of high-performance composite materials, green manufacturing processes, and intelligent applications.

## 1. Introduction

Basalt is a common igneous rock, formed when magma in the mantle is ejected to the surface by volcanic activity, which is followed by cooling and solidification [1,2]. Thus, it is a natural volcanic extrusive rock, and its main components are pyroxene, plagioclase, and olivine, with high iron and magnesium contents and a relatively low silicon content [3,4,5]. For centuries, natural stone basalt has been extensively used as a raw material for constructing houses, roads, river walls, and dams due to its high strength, durability, skid resistance, and drainage properties. About 100 years ago, the ground basalt raw material was melted at 1300 °C and then cast into bricks, slabs, and other building materials [6]. It was not until 1922 that Paul Dhe [7,8] first proposed and described the manufacturing technology of filament made of basalt in a patent. Since then, many patents on the production process and properties of basalt fiber (BF) have gradually been published. After 1960, BF became a major research target for military use in the United States and the former Soviet Union. By the 1970s, the research and development focus of American companies shifted to glass fiber (GF) and ceramic fiber, while the focus of the Soviet Union on BF remained high. In the late 1990s, the Soviet Union successfully developed a new-generation production process for BF, with significantly reduced energy consumption [9]. Nowadays, the research on BF, its production, and sales is mainly carried out in countries such as Russia, Ukraine, and China [6,7].

**Figure 1:**
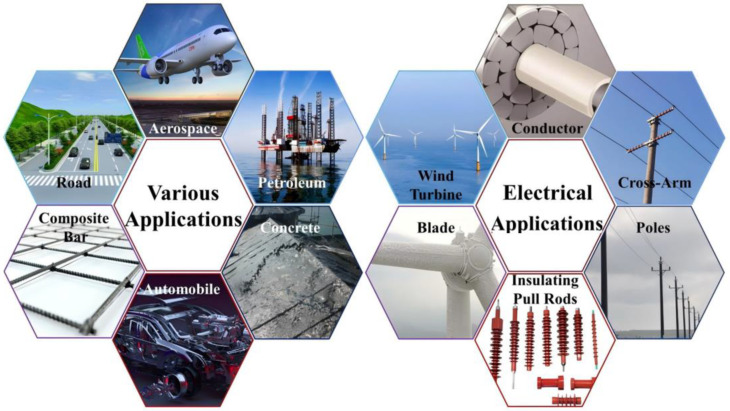
Applications of BF in various sectors [9].

BF is a type of fiber with high strength, excellent modulus, high temperature resistance, cost-effectiveness, and non-toxicity [10]. It is natural, environmentally sustainable, easy to manufacture, and an effective green material for environmental protection [11]. Figure 1 shows that BF has been widely used in various industries. In the construction industry, BF has been utilized extensively as concrete reinforcement and external-wall insulation material, attributed to its high strength, corrosion resistance, and high-temperature stability. In the aerospace field, the high specific strength and modulus of BF make it an important component of light-weight composites. In the automobile industry, its application not only reduces the weight of the vehicle refining the fuel efficiency but also improves the impact resistance of the vehicle. In the energy sector, BF has been used to reinforce wind turbine blades, leading to a significant extension of their service life [9,12,13,14].

In recent years, BF has been widely used as a reinforcement material in the preparation of fiber-reinforced resin composites (FRRCs) [15,16,17]. The mechanical properties of these FRRCs are primarily impacted by fiber properties, resin characteristics, and interfacial bonding strength between the two. However, as an inorganic reinforcement, the surface structure of BF is relatively smooth, lacking functional groups. BF cannot interact with the matrix, and its interfacial bonding strength with the polymer matrix is relatively weak. Moreover, BF can easily slide from the fiber–matrix interface, resulting in an insufficient carrying capacity of FRRCs [18,19,20]. To avoid debonding, pulling out, fiber sliding, and crack bridging phenomena that may occur at the fiber–matrix interface, good adhesion or sufficient physico-chemical interaction between the fiber and the matrix should be achieved. These phenomena significantly impact the total energy consumption during crack propagation and can be controlled by fiber reinforcement [21]. Therefore, when BF is used to reinforce polymer to prepare composites, it needs to be surface-treated to improve its adhesion and dispersion [22].

In recent years, with the improved interest in environmental awareness and the growing demand for new materials, the studies and applications of BF have made significant progress [5]. The preparation technology, performance optimization, and application development of BF have been extensively studied [23]. A variety of modification techniques—such as chemical surface treatment, plasma treatment, and nanomaterial composites—have been used to enhance the interfacial bonding strength and mechanical properties of BF [24,25]. Moreover, the production process of BF is relatively environmentally friendly, and it is in line with the development trend of green materials, which has attracted widespread attention [22,26]. With the increase in awareness toward the environment, the number of reports and fabrication methods of environmentally friendly materials around the world have exploded, which has renewed the attention toward the potential applications of BF. Development of economical, high-performance BF-reinforced resin matrix composite (BFRRC) is urgently required to meet the needs of multiple engineering and industrial applications. Therefore, more research attention should be paid to improvements in the fabrication techniques of BFRRC, and their performance optimization should be further explored. Therefore, as a starting point, outlining the context of BFRRC is helpful.

The preparation technology, properties, and parameters of BFRRC and surface treatments are presented in this review. The influence of non-destructive surface-modification methods on the properties of BFs is emphasized. The research progress and application effect of BF surface-modification methods are also introduced. The main objective of this review is to provide references and insight into the further research and application prospects of BFRRCs through the analysis and summary of the relevant literature and to provide new ideas and inspirations for researchers in related fields, such as construction, automotive, aerospace, and power.

## 2. Production Process and Properties of BFs

**Figure 2:**
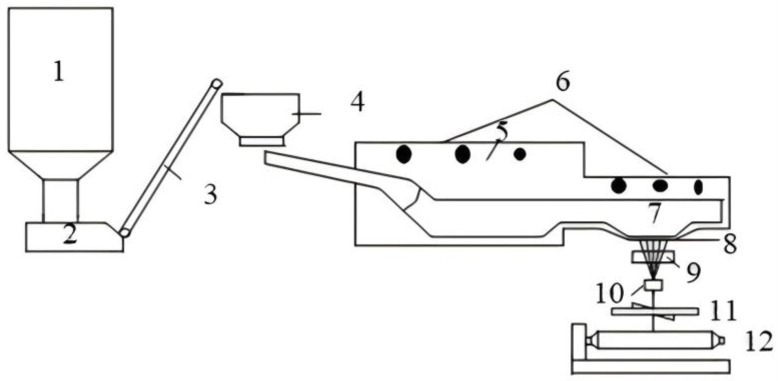
BF production process flow: 1—silo; 2—feeder; 3—lift conveyor; 4—quantitative feeder; 5—primary melting zone of raw materials; 6—gas nozzle; 7—secondary melting belt (front furnace); 8—platinum–rhodium alloy leakage plate; 9—application of infiltrating agent; 10—buncher; 11—fiber tensioner; 12—automatic winding machine.

**Figure 3:**
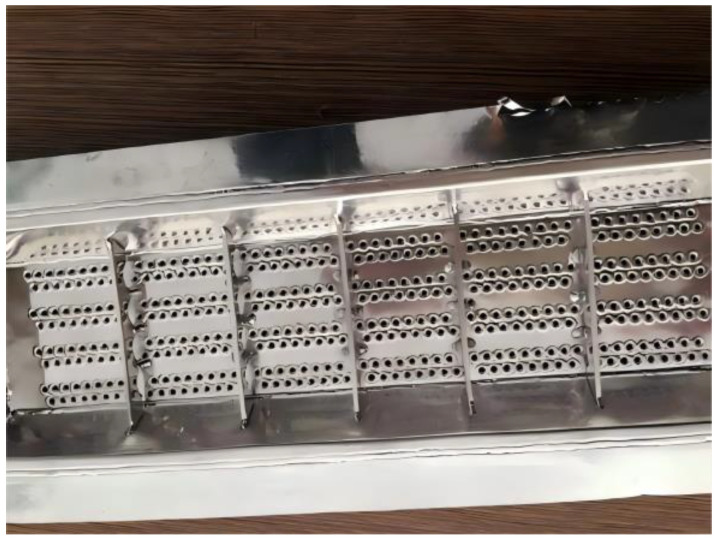
Platinum–rhodium alloy bushing plate.

Figure 2 exhibits the production process of BFs. Briefly, BF is prepared by melting the washed acidic basalt (silica > 46%, white) at about 1400 °C. The mineral composition of basalt mainly consists of anorthoclase (Na(AlSi_3_O_8_)–Ca(Al_2_SiO_8_)), pyroxene (XY[(Si,Al)_2_O_6_] (where X represents Ca, Mg, and Fe^2+^, and Y represents Fe^3+^, Al, and Ti)), and olivine ((Fe,Mg)_2_ SiO_4_). The rheological properties of basalt melts—primarily viscosity and its behavior, surface tension, density, and crystallizability—aid in determining the fundamental possibility of them forming a continuous fiber from melt [27]. The molten basalt is extruded into continuous filament fibers with a platinum–rhodium alloy bushing plate (Figure 3) under hydrostatic pressure. The high viscosity and low surface tension of the melt make the production process very stable, non-toxic, and non-carcinogenic [28].

**Figure 4:**
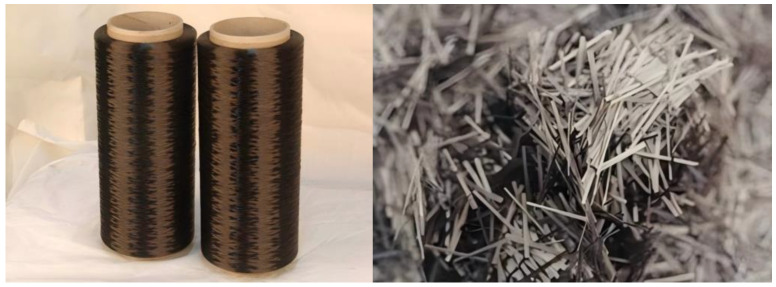
Photographs of BFs ((**left**): filament fiber, (**right**): chopped fiber).

After the wire drawing, a cutting machine can be used to further chop the produced continuous BFs for manufacturing the desired composite material [29]. Figure 4 shows the photographs of BFs, with the diameter in the range of 10–20 μm. The short-cut BFs offer the advantages of changing geometric parameters (i.e., diameter and length) and random orientation.

Moreover, BF is non-combustible, chemically stable, and highly resistant to weather, alkali, and acid exposure [30,31]. It can be used from very low temperatures (i.e., about −200 °C) to high temperatures in the range of 600–800 °C [32].

BF is also biologically inert and naturally resistant to ultraviolet (UV) and high-energy electromagnetic radiations; thus, it can be used to fabricate low-cost yet high-strength composites for application in harsh environments [30,33].

Table 1 presents a detailed comparison of the thermal properties of BFs and GFs. The thermal properties of BFs are better than those of GFs, thus making BFs more suitable for heat-resistant materials.

Table 2 presents a comparative analysis of the electrical properties of BFs and electric-glass fibers (E-GFs), revealing that the electrical properties of the two types of fibers are similar.

The chemical properties of BFs and GFs are listed in Table 3. The alkali and acid resistance of BFs is better than that of GFs, and alkali solution is more corrosive to the BF surface than acid solution.

In summary, compared with GFs, BFs exhibit better comprehensive properties and are more suitable as reinforcing material for resin matrix composites. However, the smooth surface and chemical inertness of BFs weaken their adhesion ability to the resin–matrix interface. Therefore, it is necessary to improve the interfacial property by surface modification to improve the comprehensive properties of the resulting composites.

## 3. Basalt-Fiber-Reinforced Resin Matrix Composites

The choice of matrix material significantly impacts the performance of composites. The commonly used matrix materials for BFRRCs are thermosetting resin and thermoplastic resin. The heat resistance and rigidity of thermosetting resin are higher than those of the thermoplastic resin, and the price is lower than the thermosetting resin. Nonetheless, the thermoplastic resin exhibits good fracture toughness and can be repeatedly melted and reused. Moreover, thermoplastic resin is not resistant to organic solvents, while thermosetting resin can usually resist most organic solvents [38,39,40,41].

Table 4 presents the data that highlight the differences in the mechanical properties of the resin matrix and the BFRRCs. After adding BFs, the tensile strength, flexural strength, bending strength, and impact strength of the resin matrix increased by 2626%, 804%, 70%, and 339.3% at the highest, respectively. BFs exhibited the best effect on improving the mechanical properties of unsaturated polyester resin (UPR), followed by epoxy (EP) resin, polyethylene (PE) resin, polypropylene (PP) resin, and polyamide (PA) resin. BF-reinforced resin matrix can significantly improve the mechanical properties of the resin matrix; therefore, BFRRCs are usually more likely to interest the manufacturers than resin.

### 3.1. BF-Reinforced Thermosetting Resin Composites

Thermosetting resin is cured with a heating or curing agent and formed into an irreversible three-dimensional (3D) network structure [47]. It shows excellent bonding, strength, corrosion resistance, and aging resistance and is an important matrix material for high-performance composites [48]. Commonly used thermosetting resins include EP, UPR, and phenolic (PF) resins.

#### 3.1.1. BF-Reinforced EP Resin Composites

EP resin exhibits excellent bonding, high mechanical strength, and good chemical corrosion resistance [49]; thus, it has become one of the most widely used thermosetting resins. EP resin is extremely suitable as the base material of BF for fabricating BF-reinforced EP resin composites (BFREPCs). Previous related studies have reported the mechanical properties, thermodynamic properties, electromagnetic shielding properties, and durability of BFRECs to more comprehensively evaluate the performance of BFREPCs and reveal the property enhancement effect of BFs on BFREPCs [50,51,52,53,54].

1. Mechanical properties

**Figure 5:**
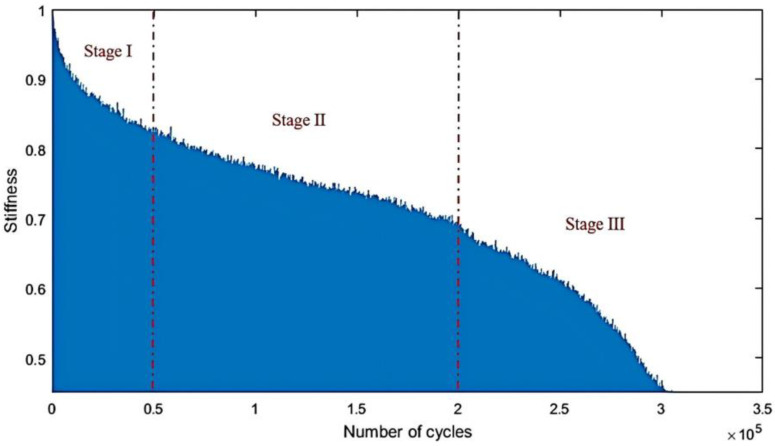
Three distinct stiffness reduction zones in the history of fatigue sample [50].

The mechanical properties of the materials include strength, stiffness, toughness, and fatigue performance. The mechanical properties of BFREPCs directly affect their use, effect, and service life, thus gaining significant research attention from scientists and engineers at the global scale. For instance, Chhorn and Jung [50] investigated the flexural fatigue performance of BFREPCs and reported that under fatigue loading of 122.24 MPa, the stiffness degradation process of composites could be divided into three stages (Figure 5). The first was the high rate of stiffness degradation at the first few thousand cycles. The second stage then occurred with gradual stiffness degradation, covering a sizeable portion of the component’s life. Finally, more grave types of damage—such as fiber fracture—occurred that induced complete material failure. Chowdhury et al. [51] analyzed the failure modes of BFREPCs; the study revealed that for ex situ flexure and in situ tests, the damage was initiated on the compression side at the top ply. The damage was initiated in the form of fiber/matrix debonding and led to crack generation in the matrix in the 90° sub-ply and delamination at the interface between 0° and 90° sub-plies. Final failure was found to be associated with fiber kinking in the 0° sub-ply. Bozkurt and Gökdemir [38] found that with the increase in the volume content of BFs, the vibration-damping performance of BFREPCs improved significantly.

The main factors affecting the mechanical properties of BFREPCs are the strength of BFs, the mechanical properties of the EP resin, and the interfacial bonding strength of the BFs and EP resin. On the premise of determining the types of BFs and EP resin, the mechanical properties of BFREPCs can be significantly improved by increasing the interfacial bonding strength between the BFs and EP resin. To improve the interfacial bonding strength between the BFs and the EP resin matrix, Preda et al. [55] functionalized BFs by zinc oxide (ZnO) electroless deposition. They obtained a nanostructured interphase, which resulted in enhancement in the interfacial shear strength by 42%. Mittal et al. [20] coated graphene oxide (GO) on BFs through electrophoretic deposition (EPD), and modified BF (MBF) was further used to reinforce EP. The effects of MBF on the mechanical and tribological performance of BFREPCs were investigated. Results showed that the improved fiber–matrix bonding and uniform distribution of GO led to enhancement in the bonding of BFs to the EP resin matrix. The above-mentioned studies indicate that coating BFs by EPD is an effective method to improve the interfacial bonding strength between the BFs and the EP resin matrix. Moreover, the BFREPCs prepared with MBF show better tensile strength and less wear loss.

2. Thermodynamic properties

As composites, BFREPCs are often used in multiple scenarios, such as high-temperature and acid–base erosion environments. The thermodynamic properties of BFREPCs are critical in high-temperature environments. To prepare BFREPCs with excellent thermodynamic and flame-retardant properties, the thermodynamic properties of BFREPCs are improved via BF modification.

Kim et al. [52] studied the electric heating performance of EP composites reinforced with multi-walled carbon nanotube (MWCNT)-coated BFs. The results indicated that the composites prepared with MWCNT-coated BFs fabricated by two cycles of dip-dry coating exhibited excellent electric heating performance in terms of rapid temperature responsiveness, high steady-state maximum temperature, high electric power efficiency, and stable operational stability under applied voltages. Guo et al. [56] introduced MBF into the flame-retardant EP composites (EP/AP750) to prepare BF-reinforced flame-retardant EP composite (EP/AP750/BF-AT). Compared with the flame-retardant properties of EP/AP750, the incorporation of BF-AT led to a slight reduction in the limiting oxygen index (LOI) of EP/AP750/BF-AT from 26.3% to 25.1% but increased the peak of the heat release rate of EP/AP750/BF-AT in the vertical burning test (ASTM D 3801-10) [57]. Karvanis et al. [58] prepared BFREPCs using EP resin as the matrix by a hand lay-up compression molding combined method and analyzed the thermodynamic and kinetic properties of BFREPCs. The results showed that with the increase in loading frequency during dynamic mechanical analysis (DMA), the glass transition temperature of BFREPCs increased. When the temperature range was 30–900 °C, the BFREPC did not decompose in nitrogen or air. The study proved that BFREPC exhibits excellent high-temperature resistance and can be used as a fire insulation material.

3. Electromagnetic shielding properties

The application of BFREPCs in the medical industry has also gained significant research attention because of the excellent electromagnetic shielding properties of BFREPCs. For instance, Dhand et al. [53] prepared eutectic-Bi–Sn-coated BF-reinforced carbon nanotube (CNT)/EP hybrid composites. The material could effectively manage and shield clinical X-ray radiation and electromagnetic interference (EMI), with a total shielding effectiveness of 34.4 dB. The mechanistic study showed the synergistic effect between Bi–Sn coating and CNT filler. Mittal et al. [59] prepared hierarchical structures of BFs coated with CNTs (BF-CNTs) by chemical vapor deposition (CVD) and analyzed their effects on the tribological and electrical properties of EP composites. The results showed that the BF-CNT composites prepared at higher growth temperatures and for longer growth times exhibited a lower coefficient of friction, lower wear loss, lower volume resistivity, and improved electromagnetic interference shielding effectiveness (EMI SE).

4. Durability

The durability of BFREPCs aids in determining the service life and use cost of BFREPCs. However, relatively few studies have been reported on the durability of BFREPCs to date. To solve the aging problem of BFREPCs in seawater environments, Ulus et al. [54] introduced halloysite nanotubes into an EP resin matrix to improve the fiber–matrix interface. This method not only increased the mode II delamination toughness and shear strength of BFREPCs by 46% and 34%, respectively, but also increased the glass transition temperatures of BFREPC by 8%, which effectively improved the anti-aging property of BFREPCs in marine water.

The above-mentioned results show that the incorporation of BFs leads to improvement in the mechanical properties, thermal properties, electromagnetic shielding properties, and durability of the resin. The excellent properties of BFREPC make it suitable for the preparation of a variety of materials, such as fatigue-load-resistant materials, vibration-damping materials, heat-resistant materials, flame-retardant materials, and electromagnetic shielding materials. However, to date, relatively few studies have reported on the durability of BFREPCs when used in different environments. To obtain composites with good comprehensive properties, the durability research on BFREPCs should not be ignored in subsequent studies.

#### 3.1.2. BF-Reinforced UPR Composites

To note, the cost of polyester is lower than that of EP resin, and its mechanical properties and weather resistance are better. Moreover, polyester is suitable for manufacturing large sizes and complex shapes of composites [60]. Types of polyester resin include saturated polyester resin (PR) and UPR, and UPR is a cost-effective matrix material with good bonding properties.

1. Mechanical properties

**Figure 6:**
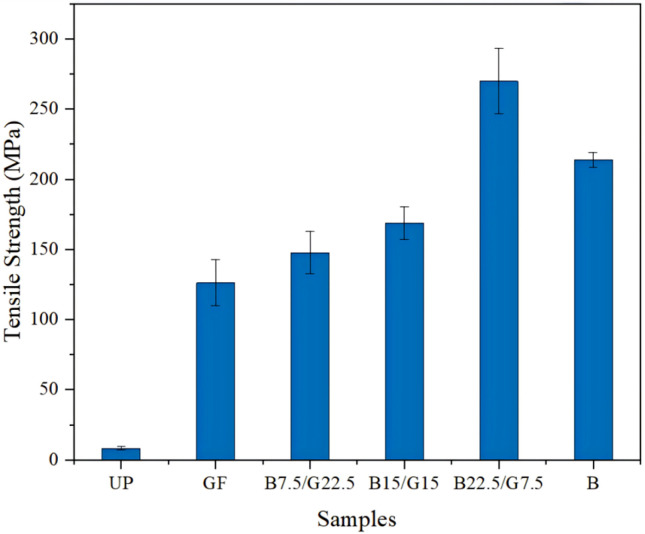
Tensile strength of basalt/glass hybrid composites [43].

To prepare BF-reinforced UPR composites (BFRUPCs) with excellent mechanical properties, in the previous studies, researchers used a variety of fibers to reinforce BFRUPCs and determined the best raw-material ratio for BFRUPCs by comparing the performance differences among different BFRUPCs. Sapuan et al. [43] investigated the mechanical properties of longitudinal basalt/braided GF-reinforced UPR composites (GFRPCs). The results showed that the addition of BFs to GFRPCs could improve the density, tensile properties, and flexural properties. Figure 6 exhibits that the tensile strength of B22.5/G7.5 (the dosage of BF and GF was 7.5 and 22.5 wt.%, respectively) hybrid composites increased by 213.92 MPa compared with neat UP, which was 8.14 MPa. The study proved that the mechanical properties of blended-fiber-reinforced resin composites are superior to those of single-fiber-reinforced resin matrix composites. Prasath et al. [61] studied the effect of different stacking sequences of flax fiber (FF) and BFs on the low-velocity-impact and compression-after-impact responses of reinforced UPR composites. They found that the alternate arrangement of BFs and FF composites showed better performance in terms of low-velocity impact and compression. Manikandan et al. [62] studied the effects of BFs treated with and without acid–base treatment on the mechanical properties of BFRUPCs, including tensile, shear, and impact strengths. The results showed a change in the surface of the BFs treated with acid and alkali and an improvement in the adhesion of BFs and the matrix. Furthermore, it was found that the mechanical properties of BFRUPCs are better than those of GFRPCs. The study confirmed the applicability of BFs as a reinforcing agent in composites. Chairman et al. [39] found that the bending strength, interfacial bonding properties, and wear resistance of BFREPCs were superior to those of BF-reinforced polyester resin composite.

**Figure 7:**
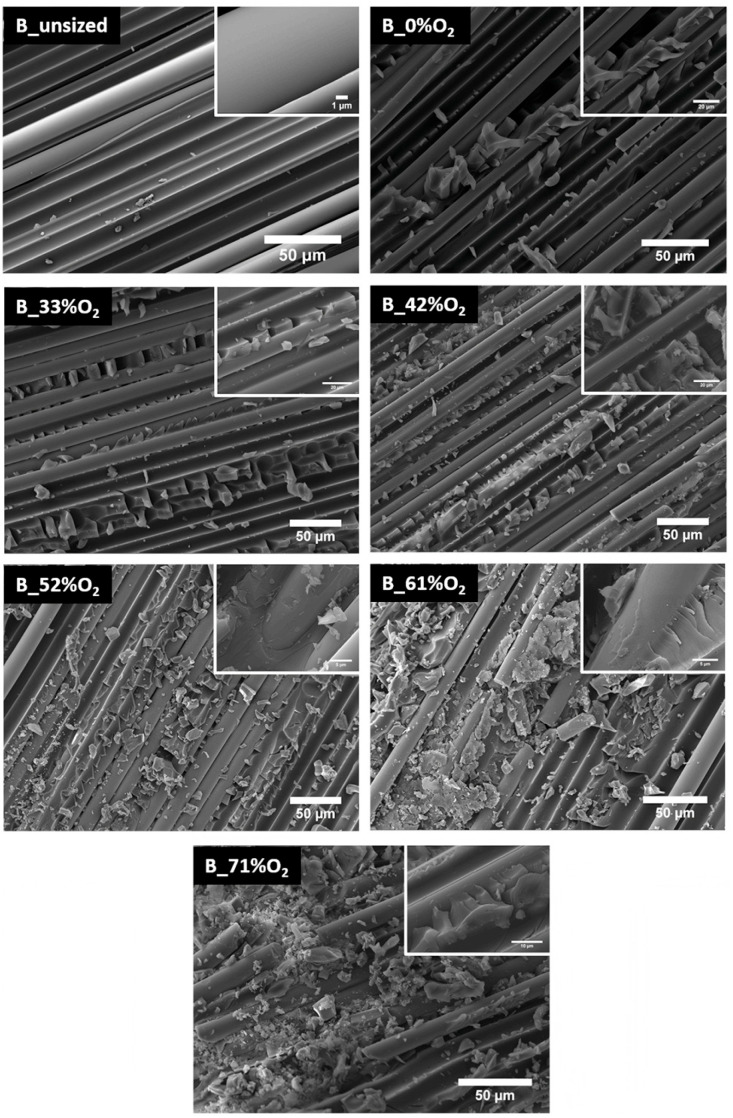
SEM micrographs detailing the fracture morphology of BF/polyester composite beams for unsized basalt fibers and basalt fibers after polymerization processes. “B” stands for basalt fiber, while the percentage refers to the oxygen fraction in tetravinylsilane mixture [63].

The interfacial bonding properties of the fibers and the resin matrix have been widely explored by researchers. To solve the problem of insufficient interfacial bonding strength between BFs and the resin matrix, Lilli et al. [63] used a plasma polymerization technique to synthesize a layer of polymeric coating on the BF surface. The polymer coating was either tetravinylsilane or its mixtures with oxygen. The results showed that the interlaminar shear strength (ILSS) of MBF increased by more than 180% and improvement in the interfacial adhesion of MBF and UPR was also observed. As can be observed in Figure 7, after the plasma polymerization process, the number of matrix layers attached to the surface of the BFs increases, thus suggesting a stronger interfacial adhesion [63].

2. Comprehensive properties

Notably, it is still required to improve the comprehensive properties of BFRUPCs due to the low strength of the bio-based material itself [64]. Therefore, researchers have tried to improve the overall properties of BFRUPCs by mixing enhancers. For instance, Jevanantham et al. [65] improved the load-bearing, time-dependent, and thermal properties of BFRUPCs by adding peanut-shell-derived silicon nitride (Si_3_N_4_). The results showed that the addition of Si_3_N_4_ exhibited little effect on the density and water content of BFRUPCs; however, it could effectively improve the wear resistance, flame retardance, thermal stability, and tensile strength of BFRUPCs. The BFRUPCs with Si_3_N_4_ showed a specific wear rate of 0.009 mm^3^·Nm^−1^, a friction coefficient of 0.31, and a low creep strain of 0.0086 mm within 10,000 s. At the high-initial-decomposition temperature of 410 °C and low-flame-propagation speed of 6.72 mm·min^−1^, tensile strength was up to 130 MPa. Dayalan et al. [66] prepared composites by mixing BFs, GFs, and Hemp fibers (HFs) with UPR, and investigated the effects of the addition of nano silica on the mechanical properties and water-absorption characteristics of the composites. The results showed that the mechanical properties of the composites first increased and then decreased with the increase in the content of nano silica. When the content of nano silica reached 2%, the mechanical properties of the composites were optimal. The water absorption behavior study showed that until the water uptake percentage attained a constant value, the equilibrium water uptake decreased until 3% of nano silica loading.

Moreover, extensive research efforts have been devoted to the study of the mechanical properties of BFRUPCs. Although researchers have previously studied the overall properties of BFRUPCs, the investigation was not comprehensive, and only two or three properties of BFRUPCs were studied simultaneously. In follow-up research, an investigation of the comprehensive properties of BFRUPCs is required to further expand the application scope of BFRUPCs.

#### 3.1.3. BF-Reinforced PF Resin Composites

PF resin is a type of high-performance ablative resin, with the advantages of a high carbonization rate, compact and uniform carbonization layer, and low ablative rate [67]. However, PF resin possesses certain disadvantages, including high brittleness, poor toughness, and low shear strength after curing [68,69]. Nonetheless, the high toughness of the fiber materials can make up for the shortcomings of PF resin. Therefore, PF resin is often used as a matrix for BFRPFCs.

1. Mechanical properties

To further expand the application scope of BFRPFCs, the mechanical properties of medium-density fiberboard (MDF), brake pads, anti-fatigue materials, and BFRPFC-reinforced cement-based materials have been comprehensively investigated. The research has proven that BFRPFC exhibits excellent mechanical properties in a variety of materials.

**Figure 8:**
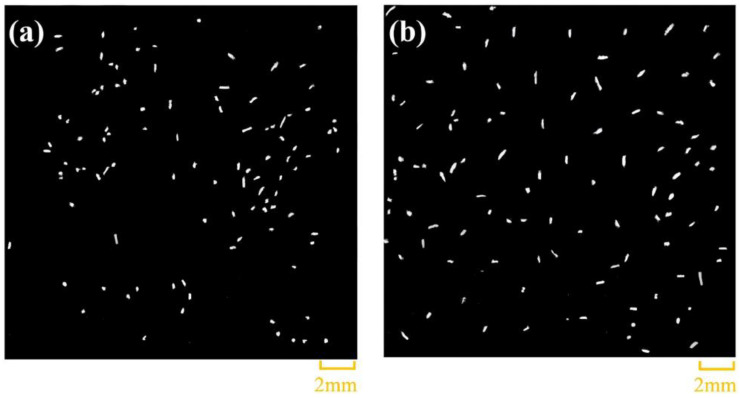
SEM binary processing images of (**a**) BF and (**b**) MBF in cement [73].

Pugazhenthi and Anand [70] prepared MDF using coir fiber, BFs, and PF resin. When the volume ratio of coir fiber–BF–PF resin was 5:3:2, the internal bonding strength of MDF reached the maximum value of 2.1 N·mm^−2^, and the modulus of rupture (MoR) and modulus of elasticity (MoE) reached 29.14 and 3143 N·mm^−2^, respectively. The study strongly proved that the coir fiber and BFs can be used together to produce the hybrid MFD, which can help engineering wood manufacturers to produce hybrid boards using coir fiber and BFs. Jacob Moses et al. [71] used BFs and GFs instead of asbestos to prepare a new type of brake pad, and studied its physical properties and wear behavior. They found BFs and GFs to be particularly suitable reinforcement materials for brake pads [72]. Li et al. investigated the effect of p-t-octylphenolic resins on the adhesion between continuous BFs (CBFs) and a natural rubber (NR)/styrene–butadiene rubber (SBR) matrix. The results showed that p-t-octylphenolic resins could effectively improve the static adhesion and interfacial fatigue life of CBFs and the matrix. To improve the dispersion of fiber in cement base, Peng et al. [73] used phenolic epoxy resin as the raw material and modified BFs with methacrylic acid and coupling agent KH-570. A hydrophilic cationic coating agent was obtained by adding cetyltrimethylammonium chloride, and it was coated onto the surface of BFs to obtain MBF. MBF had a 27.2% stronger adhesion than before modification, and its distribution coefficient increased by 73%. Figure 8 demonstrates the significant improvement in the dispersion of MBF in the cement matrix. MBF cement showed compressive and flexural strengths that were, respectively, 15.9% and 13.5% stronger than those before modification.

2. Thermodynamic properties

To date, many systematic explorations have been carried out on a wide range of the thermodynamic properties of BFRPFCs to improve the ability of BFRPFCs to resist high-temperature environments.

To avoid the use of carcinogenic asbestos fiber, Kumar et al. [74] used BFs and FF instead of asbestos fiber as the reinforced material in friction material composites. The results showed that the specimens reinforced with a 6% volume fraction of FF and BFs exhibited good thermal stability compared with the other volume fractions. Reza Eslami-Farsani et al. [75] investigated the effect of thermal cycling on the hardness and impact-resistance of carbon/PF (CFP)-, basalt/PF (BFP)-, and basalt/carbon/PF (BCFP)-reinforced PF resin composites. They found that among the three composites, the BCFP composite exhibited the best impact resistance, and its impact energy was about 190% higher than that of the CFP composite. The study further proved the performance advantage of mixed-fiber-reinforced resin matrix composites in high-temperature environments. Further, Li et al. [76] investigated the thermophysical and thermomechanical properties of PF–BF-reinforced polymer (P-BFRP) rebars at elevated temperatures and compared them with those of vinyl BFRP (V-BFRP) and EP-BFRP (E-BFRP) rebars. They found that the glass transition temperature of P-BFRP rebars was significantly higher than that of the other two types of BFRP rebars. This advantage of P-BFRP rebars was mainly attributed to the significantly higher glass transition temperature, which delayed the initiation of interfacial bonding failure up to 300 °C. Therefore, P-BFRP rebars exhibited superior tensile strength compared with V- and E-BFRP rebars under high-temperature conditions. The high tensile strength of P-BFRP rebars in the range of 100–300 °C could significantly extend the fire-resistance duration in the case of an applied fire protection system. However, Lohmus et al. [77] found that the reinforcement effect of the BFs and the PF resin on plywood was completely lost at 150 °C and recovered after slowly cooling it down back to room temperature (24 h). The deterioration was attributed to the weakening of bonding between the BFs and the PF resin matrix at elevated temperatures due to the resin softening. Thus, different conclusions have been reported on the thermodynamic properties of BFRPFCs. Therefore, in the future, further research should focus on an in-depth study of the thermodynamic properties of BFRPFCs.

Furthermore, many systematic investigations have been carried out on the mechanical properties, thermodynamic properties, electromagnetic shielding properties, comprehensive properties, and durability of BFRRCs. According to the literature, the key factors that restrict improvement in the properties of BFRRCs are as follows:

1. The weak BF–resin interface. The weak BF–resin interface limits the application of BFRRCs. Particularly, in the extreme environment of high temperatures and erosion, unsticking often occurs between the fibers and the resin matrix, leading to disruption in the adhesion between them. In general, the fiber is usually modified to improve the interfacial bonding strength between the fiber and the resin, which improves the performance of BFRRCs. However, it is often difficult to achieve fiber modification in large-scale production due to the complex operation process and high cost. Moreover, attention should be paid to the feasibility of BFRRCs in actual production processes in subsequent studies;
2. The increase in single-fiber content shows their limited contribution to improving the properties of BFRRCs. A mixture of various fibers can improve the mechanical properties, impact resistance, and wear properties of composites. In general, the main objective of preparing composites by blending fibers is to reduce the cost of composites without reducing their properties. Characteristics of BFs such as high strength, low cost, and environmental protection make them particularly suitable for use as blending fibers; consequently, the composites can be prepared with certain economic and environmental benefits, and the process is conducive to the further promotion and use of FRRCs [11,61].

### 3.2. BF-Reinforced Thermoplastic Resin Composites

Thermoplastic resin is a class of polymer materials that softens when heated and re-hardens when cooled. It undergoes physical rather than chemical changes when heated to a certain temperature, allowing it to maintain its properties over multiple heating and cooling cycles. This property increases the widespread application of thermoplastic resins in industry and daily life [78].

Compared with thermosetting resins, thermoplastic resins show excellent fracture toughness and anti-fatigue behavior and are widely used as a matrix material in FRRCs [79,80,81]. PE, PP, and PA have been widely used as the thermoplastic matrix of BFs.

#### 3.2.1. BF-Reinforced PE Resin Composites

PE resin is a thermoplastic resin prepared by polymerization using ethylene as the starting material. It exhibits excellent low-temperature resistance (the use temperature can reach −70 to −100 °C), good chemical stability, resistance to most acid and alkali erosion, and excellent electrical insulation properties [82,83]. However, PE is very sensitive to environmental stress (chemical and mechanical effects) and exhibits poor heat resistance and aging performance [83]. Therefore, modification and reinforcement of the PE resin are essential for its application.

1. Mechanical properties

**Figure 9:**
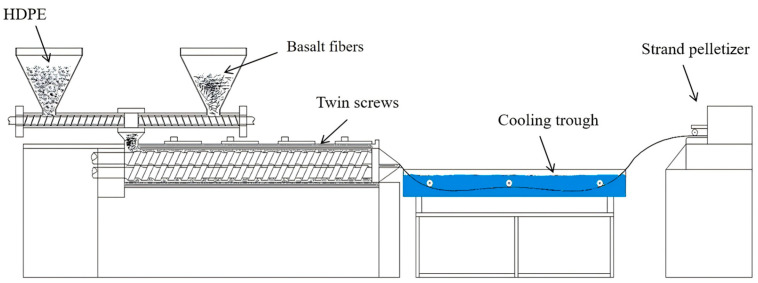
Schematic illustration of the twin screw extrusion process for manufacturing composite [45].

**Figure 10:**
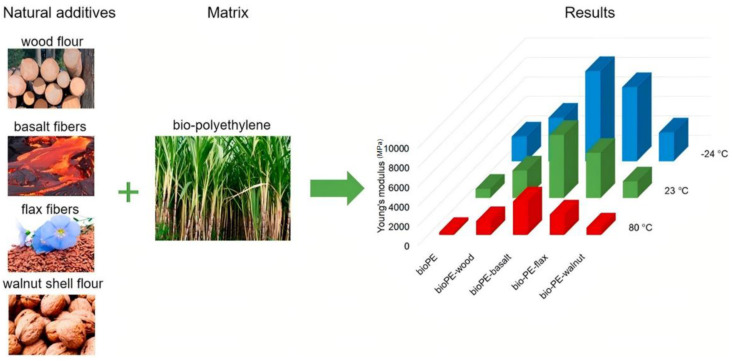
Green HDPE composites reinforced with BFs and agricultural fillers [86].

Many studies have proven that BFRPECs exhibit better mechanical properties than PE resin. For instance, Bazan et al. [44] evaluated the possibility of using natural fibers (NFs) (flax, coconut, BFs, and wood flour) as a reinforcement of bio-PE. The results of the static tensile test, three-point bending test, and impact strength test showed that the NFs enhance the strength and rigidity of the bio-PE material. Further, Bazan et al. [84] mixed coconut fiber, BFs, and wood flour in different combinations to enhance the bio-PE composites. They found that the mechanical properties of the composites could be improved by the synergistic influence between the fibers after the introduction of a variety of fibers into the polymeric matrix. Adole et al. [45] prepared BF-reinforced high-density PE (HDPE) composites by a twin-screw extrusion process (Figure 9). The results showed that the mechanical properties of the new composites exceeded 300% that of the polymer. Kramar et al. [85] reinforced plywood with BFs and polyvinyl acetate (PVAC) and reported that their addition could increase the ultimate bending load and bending stiffness of plywood in the vertical direction by 305% and 325%, respectively; moreover, the ultimate bending load and bending stiffness in the parallel direction improved by 31% and 35%, respectively. BFs and PVAC exhibited the highest effect on the impact resistance of plywood—the energy required to fracture the specimens increased by 4213% and 6150% for one and two layers of fabric, respectively, which led to significant improvement in the sample toughness. Mazur et al. [86] prepared a composite of wood flour, BFs, FF, and walnut shell flour based on bio-based HDPE (Figure 10) and evaluated the effects of NFs on the mechanical, thermal, and hydrogen degradation behavior of the composites. The results showed that the NFs enhanced the mechanical properties of composites, in particular, the stiffness of composites. After adding BFs, Young’s modulus and the tensile strength of composites increased by 600% and 156%, respectively.

The above-mentioned studies indicate that the mechanical properties of BFRPECs, such as strength, stiffness, and toughness, are superior to those of PE resin. Moreover, BFs exhibit the best effect on composites in the comparative experiment on PE resin composites reinforced with a variety of fiber materials.

2. Thermodynamic properties

Further, for a comprehensive evaluation of the properties and characteristics of BFRPECs, the thermodynamic properties of BFRPECs have been extensively investigated. It has been proven that BFRPECs show better thermodynamic properties than PE resin matrix.

Zhou et al. [87] prepared BFs and PA6-reinforced HDPE composites. Further, they investigated the effects of adding fiber, organic filler, and polar component maleic anhydride (MAH) on the microstructural characteristics of the composites. The results showed that PA6 and BFs as nucleating agents could improve the crystallization rate of composites during cooling. Moreover, the thermally conductive BFs and other components could form a thermally conductive network and channels, which led to an increase in the heat resistance of the composite material. Huang et al. [88] prepared wood–plastic composites (WPCs) using BFs, talc powder, and HDPE and characterized their mechanical, morphological, and thermal properties. The results showed that the interfacial interactions between HDPE and BFs were superior to those between HDPE and talc power. Incorporation of BFs to WPCs led to significant improvement in the thermal expansion properties, flexural properties, tensile modulus, dynamic modulus, and flame resistance of WPCs. Moreover, the tensile strength and impact strength of WPCs did not reduce significantly.

3. UV protection

The study on the effect of UV light on BFRPECs has also attracted significant research attention. For instance, Van Dinh Nguyen et al. [89] prepared HDPE/wood-flour composites with a BF-reinforced shell by a co-extrusion method and exposed it to UV weathering for 2000 h. To obtain a clear contrast, the ultraviolet absorber UV326 was added to both the shell formula and the composites. The results revealed that the shells filled with 8% and 12% BFs exhibited low lightness and color change compared with those filled with UV326 for a limited duration. The combination of BFs and UV326 revealed a synergistic effect on alleviating the photooxidation of the WPC shell layers, thus verifying the UV-shielding effect.

The above-mentioned research has proven the excellent properties of BFRPECs in terms of mechanical and thermodynamic properties. However, clearly, only the properties of BFRPECs have been studied, and the actual application effect of BFRPECs in different scenarios has been ignored. Only when the material is applied in a real-life situation does it show its actual application worth, and then many problems are encountered in the material processing starting from its own properties to its practical applications. Currently, extensive research efforts are being devoted to the study of the mechanical and thermodynamic properties of BFRPECs. Undeniably, a lot more systematic exploration will be required to focus on the practical application of BFRPECs based on their properties to expand the popularization and use of BFRPECs.

#### 3.2.2. BF-Reinforced PP Resin Composites

PP resin is a semi-crystalline thermoplastic resin, with good mechanical properties, acid and alkali corrosion resistance, and good bonding characteristics and is widely used in the preparation of FRRCs [90,91]. In previous studies, the mechanical and thermodynamic properties of BF-reinforced PP resin composites (BFRPPCs) were extensively studied, demonstrating the strengthening effect of BFs on PP resin.

1. Mechanical properties

To further improve the mechanical properties of BFRPPCs, BFs mixed with other reinforcement materials are often used to jointly improve the mechanical properties of BFRPPCs. For instance, Song et al. [92] used the synergistic enhancement of cellulose nanocrystals (CNCs) and BFs to prepare a new type of BFRPPCs. They found that CNC-modified BFs led to a better enhancement of the PP than silane coupling agents (SCAs). When the mass percentages of the CNCs and BFs were 1% and 30%, respectively, the mechanical strength of the composite was highest, i.e., 64.31% higher than that of the PP matrix. The synergistic enhancement mechanism of CNCs and BFs on the PP is as follows: On the one hand, CNCs not only promoted the improvement in PP crystallinity by heterogeneous nucleation but also formed a wedge-shaped structure between them and BFs through hydrogen bonding to prevent the molecular movement of the PP. On the other hand, the BFs not only promoted the extrusion crystallization of the resin matrix but also the network structure formed by the appropriate content of BFs could realize the rapid transmission of external stress. Kufel et al. [93] prepared WPCs using PP as the matrix and adding different proportions of BFs and wood fiber. It was found that the BFs mixed with wood fiber improved the tensile and flexural properties of the WPCs and stabilized their thermal resistance. When the total fiber content was 20%, the linear coefficient of thermal expansion of the WPCs was reduced by nearly three times. Moreover, the study showed that the introduction of BFs into WPCs could improve the water absorption behavior of WPCs. Russo et al. [94] prepared BFRPPCs using film-stacking and compression-molding procedures. The effect of maleic-anhydride-grafted PP as a coupling agent on the modification of the PP matrix was investigated. The mechanical results demonstrated that the preliminary change in the matrix to enhance the interfacial adhesion led to samples with improved flexural performance, in particular, the strength and ability to withstand higher impact loading with respect to neat PP-based composite laminates.

BF is a natural fiber and a renewable resource, and it can easily replace GF and other synthetic fibers to prepare composites. Thus, it exhibits a good application prospect in environmental protection and the economy. Kufel et al. [95] used BF and GF to strengthen PP composites, and conducted the tensile test, three-point flexural test, and Charpy impact test on the samples at different temperatures. The results showed that when the fiber weight ratio was 20%, the tensile strength and tensile modulus of the composite increased by 306% and 333%, respectively. The study confirmed the feasibility of a partial replacement of GF with BF. Saleem et al. [96] used BF to partially substitute the amount of bast fibers in the polymer (PP and acrylate) composites. The addition of BF not only significantly improved the strength and stiffness of the composites but simultaneously increased the properties of the composites regarding energy absorption. The renewable hybrid polymer composites tested by the institute exhibited great potential to replace nonrenewable fiber-reinforced polymer composites. Further, Saleem et al. [97] compared the bast/BF hybrid composite with the bast/GFs hybrid composite. The specific flexural and tensile strengths of the BF-based composites were 32% higher than those of GF-based composites.

2. Thermodynamic properties

**Figure 11:**
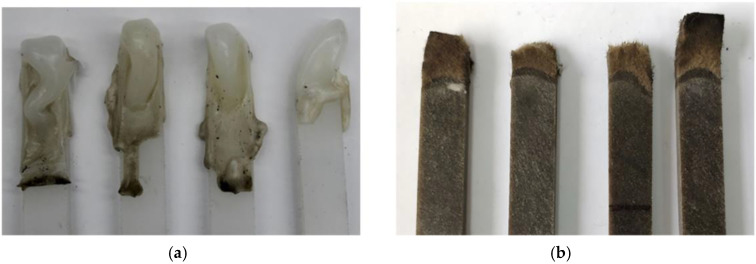
Partial photographs of samples after burning: (**a**) PP and (**b**) BFRPPC [98].

Furthermore, extensive research has also been conducted on the thermodynamic properties of BFRPPCs, and the results of the combustion test have intuitively proved that BFRPPCs exhibit better thermodynamic properties than PP. Tang et al. [98] studied and compared the thermal stability and combustion performance of BFRPPC and PP. The comparative analysis results showed that the BFs did not have any positive effect on increasing the initial decomposition temperature of PP but they could reduce the maximum thermal decomposition rate and increase the temperature of the maximum thermal decomposition rate. Figure 11 illustrates the experimental findings of combustion performance, which reveal that BFRPPC showed a better anti-melt dripping effect than PP and it was easier to ignite BFRPPC than PP. BFRPPC exhibited a lower peak heat release rate and total heat release than PP. Moreover, BFRPPC produced less smoke than PP when burned. Balaji and coworkers [24,99] studied the thermomechanical properties of BFRPPCs and found the properties to be related to initial fiber length, fiber content, and fiber orientation. BFs can improve the thermomechanical properties of composites. The tensile strength and tensile modulus of the composite material were 133% and 256% higher than those of PP, respectively. A further comparative analysis study showed that the addition of silane-treated BFs improved the modulus but caused an insignificant change in tensile strength due to fiber agglomeration from the desizing/resizing treatment, which also complicated the process. Matrix modification was proven to be more effective in enhancing the mechanical properties of composites compared with the fiber-desizing and silane treatments.

Comprehensive investigation of the mechanical properties of BFRPPC indicates that the mechanical properties of BFRPPC prepared with BF-reinforced PP resin are often limited and it is difficult for them to meet the application scenarios with the high requirements for the mechanical properties. Therefore, the researchers further advanced the mechanical properties of BFRPPC by using mixed enhancers as reinforcement, mixed-fiber reinforcement, and resin matrix modification, demonstrating that these three ways lead to obvious improvements in the mechanical properties of BFRPPC. However, few researchers have used all three of the above-mentioned enhancement methods to improve the mechanical and thermodynamic properties of BFRPPC. Thus, it is required to expand the use of BFRPPC in extreme environments such as extremely high temperatures, extremely low temperatures, and high acid–base erosion.

#### 3.2.3. BF-Reinforced PA Resin Composites

PA resin is a polycondensation polymer with a –CONH structure in the molecule, which is usually obtained by polycondensation of a dibasic acid and dibasic amine. The most prominent advantage of PA resins is that the range of softening points is particularly narrow, rather than having a gradual curing or softening process like other thermoplastic resins. When the temperature is slightly below the melting point, PA resin causes rapid curing. The presence of an amino group, carbonyl group, amide group, and other polar groups in the PA resin molecule endows it with good bonding properties, which are suitable for the preparation of composites with fiber materials [100,101,102].

1. Mechanical properties

**Figure 12:**
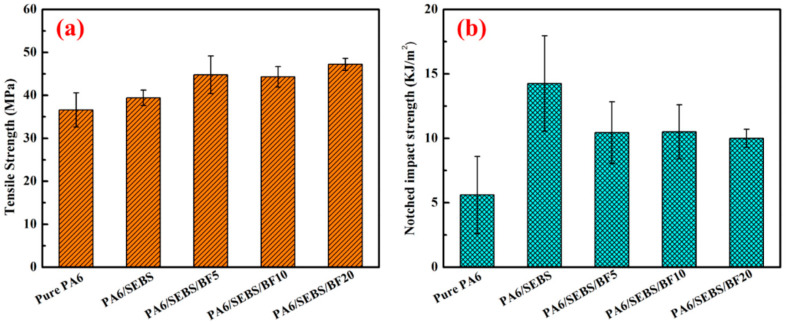
(**a**) Tensile strength; (**b**) notched impact strength of pure PA 6, PA6/SEBS blend, and PA 6/SEBS/BF composites [103].

To obtain the best mechanical properties of BF-reinforced PA resin composites (BFRPACs), the mechanical properties of BFRPACs were improved by mixing reinforcing agents and other fibers. For example, Zheng et al. [103] prepared PA6/SEBS/BF composites by melting and compounding PA6, BF, and styrene–ethylene–butylene–styrene (SEBS) copolymers, successively. As can be seen in Figure 12, the mechanical properties of PA6 improved after adding BF and SEBS. Compared with PA6, the notch impact strength of the PA6/SEBS/BF composites increased by 83%, and the wear rate of the PA6/SEBS/BF composite with added 10 wt.% BF was the lowest (2.7 × 10^−5^ mm^3^·Nm^−1^, 95% lower than that of PA6). Lee et al. [104] investigated the effects of fiber length and compatibilizing agents on the mechanical and thermomechanical properties of BFRPACs. The results showed that with the increase in BF length—because the fiber was bent by high shear force during processing—the BFs tended to be randomly oriented and dispersed more evenly, and the stiffness and thermomechanical properties of the composite improved gradually. The study provided good insights into the industrial and practical applications of long-fiber-reinforced polymer composites. Mazur et al. [46] used the injection molding method for incorporating BF and CF into PA6 to prepare the composites. The addition of BFs improved the mechanical properties and Young’s modulus of the composites, and decreased the water sorption and rate of heat emission of the composites.

Previous studies have proven that fiber modification can improve the interfacial bonding strength of the BF and PA6 matrix. Zhou et al. [105] used a sizing agent to directly coat graphene (GR) on the BF surface to prepare a multiscale graphite–BF reinforcement. The study showed that the uniform coating of GR on the surface of the BF enhanced the interfacial adhesion between the BF and the PA6 matrix; in addition, the stress transfer of the composite also improved. The tensile strength and bending strength of the composite increased by 18.2% and 34%, respectively. Yu et al. [106] used plasma polymerization to improve the interfacial bonding strength of BF and PA6,6. Thus, they could easily prepare light-weight BF/PA6,6 thermoplastic composites with high strength. They reported that plasma-polymerized BF formed a strong interface with PA6,6, demonstrating a 50.3% increase in interfacial shear strength and a 32.5% increase in tensile strength compared with untreated BF, respectively. Further, the objective was to more accurately predict the mechanical properties of BF-reinforced PA6,6 composites. Yu et al. [107] used micro-computed tomography (μ-CT) to non-destructively analyze the internal microstructure of injection-molded short BF (SBF)/PA6,6 composites and investigated the effects of fiber length and orientation on the mechanical properties of the composites. The results showed that the tensile strength of the composites prepared using the longitudinal-oriented SBF was 20% higher than that prepared by using the transverse-oriented SBF. Young’s modulus and the shear modulus of the longitudinal and transverse specimens showed slight anisotropic properties, indicating that the anisotropy of the composite system increased with fiber orientation.

GF is the most widely used fiber in FRRCs. To date, different reinforcement effects of BF and GF on the mechanical properties of PA composites have been compared and analyzed. For instance, Blackman et al. [108] added BF, GF, and talc to PA6,6 to prepare composites. They found that GF-reinforced PA6,6 showed slightly higher performance than BF-reinforced PA6,6. However, the BF-reinforced composites demonstrated better performance in tensile strength, flexural modulus, flexural strength, and heat-deflection temperature than the talc-reinforced composites. Bednarowski et al. [109] prepared GF- and BF-modified PA4.10 composites using PA4.10 as a matrix. Strength tests such as the static tensile tests, impact tests, and determination of the mechanical hysteresis loops were carried out on the composites. The results showed that composites with 30% BF composition were characterized by higher tensile strength by about 60% compared with commercially available composites with 30% GF composition and an almost doubly increased Young’s modulus. An increase in the content of BF to 50% resulted in a further increase in the strength properties. Despite the lower tensile strength compared with PA6 with 50% GF content, BF provided an approximately 10% higher modulus of elasticity. BF and GF have their own advantages, and mixing the two fibers can endow the prepared composite material with better mechanical properties.

2. Flame-retardant properties

The raw materials used for preparing BFRRCs have inherent flammability. Therefore, for ensuring the safe use of BFRRCs, the flame-retardant properties of BFRRCs have been comprehensively investigated. For instance, Barczewski et al. [110] modified PA11 using an intumescent flame-retardant system consisting of ammonium polyphosphate (APP), melamine cyanuric acid (MC), and pentaerythritol (PER), and further studied the effects of modified PA11 on the structural, mechanical, and fire behavior of BFRRCs. The obtained results confirmed the safety of using the proposed fire-retarded PA and its composites when reprocessed under the recommended process parameters, without the risk of significant changes in the structure. The study confirmed the possibility of processing fire-retarded thermoplastic polymers and their composites.

In the above-mentioned studies, the mechanical properties, thermodynamic properties, UV protection properties, and flame-retardant properties of BFRRCs were studied, and the performance differences between BF and synthetic GF composites were compared. Compared with synthetic GFs, BF is a cost-effective and renewable resource, and it offers certain environmental and economic benefits for preparing BFRRCs. However, the mechanical and thermodynamic properties of BF-based composites are lower than those of GF-based composites for different resin matrix materials. Therefore, to ensure the performance requirements of the composites, researchers usually use BF to partially replace GF in the composites, or mix other reinforcement materials to meet the specific performance requirements. With the continuous improvement in principles used for addressing environmental protection and sustainability requirements for materials, the subsequent studies should focus on the use of renewable environmental protection materials as reinforcement materials for BF, which should eventually be capable of completely replacing the synthetic materials such as GF in composites.

### 3.3. BF-Reinforced Composites Using Other Resins

Previous studies have investigated not only the resin matrix composites commonly used for BF reinforcement but also other resin matrix composites such as BF-reinforced cyanate, vinyl ester, EP vinyl, polyurethane, polybutene succinate, and polylactic acid.

1. Mechanical properties

**Figure 13:**
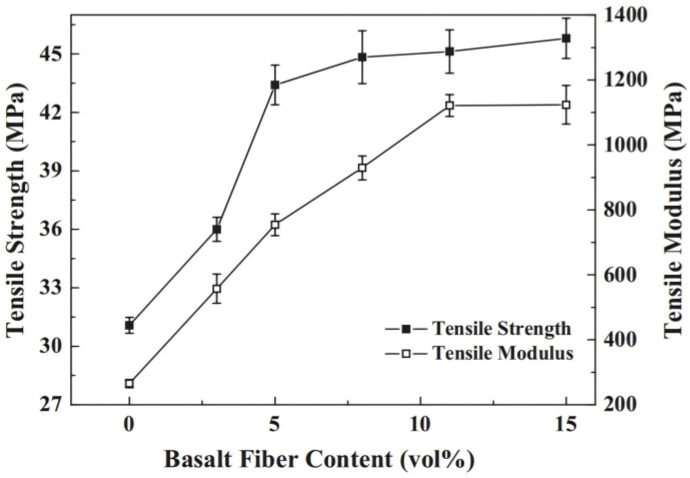
Tensile strength and modulus of BF/PBS composites with various BF loadings [114].

**Figure 14:**
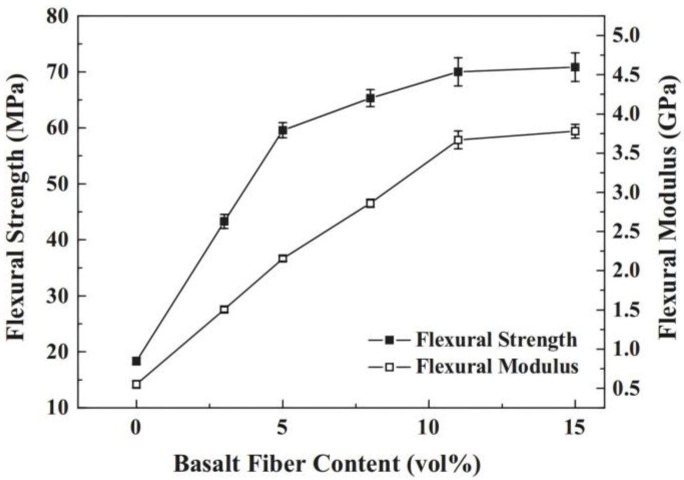
Flexural strength and modulus of BF/PBS composites with various BF loadings [114].

To reveal the influence of resin matrix on fatigue properties and the damage mechanism of BFRRCs, Zhao et al. [111] experimentally investigated the static and fatigue properties of different BFRRCs prepared using four types of resin matrix. The results revealed that the fatigue life of vinyl-ester-resin-based BFRRC was obviously lower than that of EP-resin-cured BFRRC due to more matrix cracking and fiber peeling on the surface. Although the static strength of the BFRRC prepared with a more ductile matrix such as toughened vinyl ester or room-temperature cured EP was lower, the long-term fatigue strength of BFRRC increased with an increase in the fracture elongation of the resins. Zhu et al. [112] used BF filament and vinyl ester resin as raw materials and rationally designed zigzag-shaped 3D woven spacer composites by vacuum-assisted resin transfer molding. The geometrical model of the 3D woven composite structure established by finite element simulation was compared with the experimental data of bending properties, and the results were found to be in good agreement, thus proving the effectiveness of the geometrical model. Bonsu et al. [113] prepared GF- and BF-reinforced composites using BF, GF, and epoxy vinyl ester resin as the raw materials and studied the effect of seawater treatment on the mechanical properties of the composites. They found that due to the chemical stability of BF, the tensile strength of BF composites after aging remained 100%, and the bending strength was 86.6%—the highest among all composites. Zhang et al. [114] prepared BF-reinforced poly butylene succinate (PBS) composites with different fiber contents by injection molding. They found significant improvement in the tensile and bending properties of the composites by increasing the BF content in the composites (Figure 13 and Figure 14): when the BF doping amount is 15%, the tensile and bending properties of the composite material reach the maximum values. The smaller improvement in the strength at 15 vol% may be attributed to the competitive phenomenon between the fiber reinforcement effect and micro-crack initiation as a result of the relatively high loading of basalt fibers. In practical applications, the appropriate doping amount can be selected according to the performance requirements and material cost. On the other hand, after the incorporation of BF, the heat deflection temperature (HDT) and Vicat softening temperature (VST) of the composites became significantly higher than those of the PBS resins.

To improve the interfacial bonding strength of BFRRCs, Liu et al. [115] developed three different sizing agents for BF modification. After treatment with the sizing agent, many polar functional groups—such as C=O and saturated carbon—appeared on the surface of the fibers, thus improving the adsorption and compatibility of the fibers with the resin. The modification effect was the best at the polyurethane lotion concentration of 1 wt.%. The mechanical properties of the composite, including flexural strength, flexural modulus, tensile strength, and ILSS increased by 21.05%, 31.46%, 13.89%, and 41.46%, respectively. In terms of insulation performance, the breakdown strength increased by 31.73% and the leakage current and dielectric loss factor reduced by 22.76% and 12.32%, respectively. Xue et al. [116] prepared PLA/PBAT-MDI triblock copolymers using 4,4′-methylenediphenyl diisocyanate (MDI), poly(butylene adipate-co-terephthalate) (PBAT), and poly(lactic acid) (PLA). The triblock copolymers were fused and blended to improve the interfacial adhesion of BF-enhanced PLA composite (PLA/BF). Thus, a PLA-ternary polymer with excellent properties was obtained. The results showed that the crystallinity of the PLA-ternary polymers was as high as 43.6%, 1.44 times that of PLA/BF, and 163.5% higher than pure PLA. The stored energy of the PLA-ternary polymers reached 20,306.2 MPa, 5.5 times higher than PLA/BF and 18.6 times that of pure PLA. Moreover, substantial improvement was observed in the fatigue life of the PLA-ternary polymers, i.e., 5.85 times that of the PLA/PBAT-MDI triblock copolymers. Simultaneous use of multiple materials to modify and strengthen BFRRC can endow the resulting BFRRC with better mechanical properties, which can be further explored in subsequent studies.

2. Thermodynamic properties

**Figure 15:**
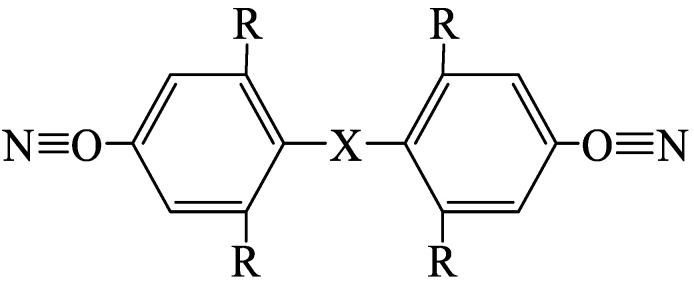
Molecular structure of CE resin monomer.

Cyanate resin is a new thermosetting resin containing two or more cyanate functional groups (–OCN) in the molecular structure and is abbreviated as CE. The general formula of the monomer molecular structure of CE resin is N≡C–O–AR–OC≡N, as shown in Figure 15, where R can be a hydrogen atom, methyl group, allyl group, etc., and X can be an isopropylidene alicyclic skeleton. CE resin exhibits good toughness, and its properties do not change with the change in frequency and temperature; thus, it can be used in a wide frequency band and temperature range [117,118]. Dreyer et al. [119] prepared composites using CE/EP polymer, BF, and CF and studied their fire reactions. The study showed considerable reductions in the heat release rate, total heat release, and CO_2_ produced for cyanate ester/EP-based composites compared with an EP-based benchmark. The synthetic fiber content and type did not significantly change time-to-ignition values but significant differences were recorded at the peak-of-heat-released rate while switching from one resin system to another. Motoc et al. [120] prepared composites using BF, CF, FF, CE, and EP resin and studied the thermal properties of the composites. They reported that the effective thermophysical properties of CF-reinforced polymer composites were better than those of BF- and FF-reinforced composites, and thermal conductivities were found to be in the range of 0.116 and 0.299 W·m^−1^·K^−1^. From room temperature to 25 °C, all composites exhibited an insulator character. BF-reinforced CE composite exhibited excellent flame-retardant properties and insulator characteristics, particularly suitable for preparing electronic materials.

The above-mentioned studies indicate that the mechanical properties, thermal stability, fatigue resistance, tribological properties, and flame-retardant properties of BFRRCs can be significantly improved. These studies have proven the feasibility of BFRRC as an effective replacement for other traditional materials such as PP or PP composites to solve the problem of environmental “white pollution”. However—attributed to biological and abiotic factors—the resin matrix of BFRRC is degradable, is easily exposed to the outside environment, and degrades and ages easily [85]. Moreover, BFRRC is often affected by a variety of environmental factors such as temperature, pH, exposure to UV radiation, and aggressive water immersion when used. Therefore, improvement in the comprehensive properties of BFRRC—in particular, the durability and safety of use—is urgently required. Further, in follow-up studies, researchers should focus on examining the comprehensive properties of BFRRC according to the complex use environment of BFRRC to broaden its application scope.

Furthermore, with increasingly severe environmental problems, at the end of the service life of composites, the recycling of composites is still a major problem. In view of this, material recycling should be considered in detail in the manufacturing stage of composites to reduce or avoid harm caused to the environment by composites at the end of their service life.

## 4. Surface-Modification Technology

**Figure 16:**
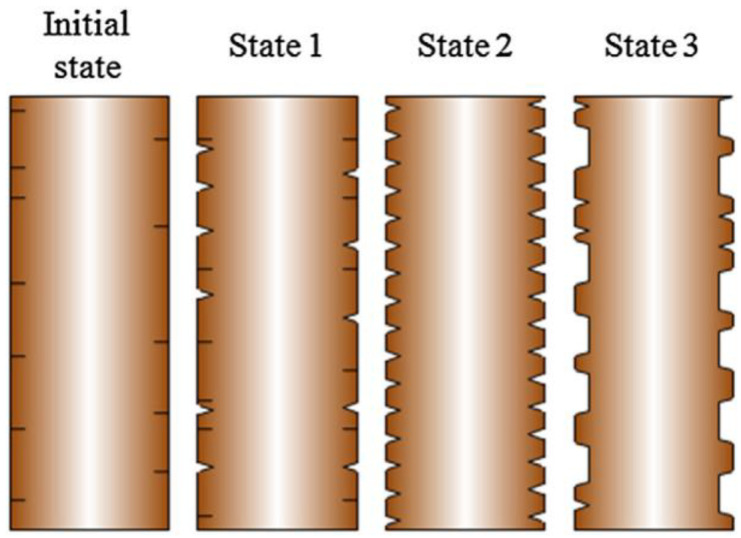
Scheme of the treatment process in H_2_SO_4_ and KOH solution [121].

To obtain BFRRC with excellent overall properties, it is necessary to further strengthen its interfacial bonding strength. In previous studies, acid–base solutions were used to etch BF, and an uneven surface topography was created to increase the anchoring ability of fibers in the resin matrix [121,122]. However, acid–base modification inevitably causes certain losses to the silicate skeleton of BF (as shown in Figure 16), thus limiting the improvement in the interfacial properties of BFRRC [121].

The plasma polymerization technique refers to the surface modification of BF by atmospheric pressure plasma such as oxygen, argon, hydrogen, or mixed gas to improve its chemical durability, surface active groups, and roughness [25,123]. However, high-temperature (above 300 °C) plasma polymerization may cause a decline in the mechanical properties of BF, and the dependence on the equipment becomes strong [63,106].

Surface modifications such as GR layer grafting and laser ablation can improve mechanical and frictional properties; however, simultaneously, the introduction of conductive GR or carbon black reduces the dielectric properties of the material. Moreover, the high cost and complex process are not conducive to the widespread use of BF in practical engineering [17,124,125].

In summary, the research and development of methods that can effectively improve the surface properties of BF without damaging its structure has become a research hotspot to make breakthroughs. In recent years, SCAs have been commonly used as BF modifiers, and new rare-earth modifiers have also been reported in some studies [19,126,127].

Table 5 presents some data on the effect of surface modification of BF on the mechanical properties of BFRRC. Notably, a variety of materials for BF surface modification, such as ZnO, GO, methacrylic acid, and CNCs, can be selected. Different BF-modified materials differently affect the mechanical properties of composites. For instance, MBF can increase the mechanical properties of composite materials by 13.2–180%. Moreover, these studies commonly reveal that MBF can significantly improve the mechanical properties of composites.

### 4.1. Silane-Coupling-Agent-Modified BF

SCAs, being organic silicon compounds simultaneously containing two functional groups, can tightly connect two types of substances with different properties, which is of great practical significance for material surface modification and material bonding [128]. Typical SCAs include KH550 (aminopropyl-triethoxysilane), KH560 (glycidyletheroxypropyl-trimethoxysilane), A151 (vinyltriethoxysilane), and A171 (vinyltrimethoxysilane).

Addition of a SCA to BFRRC can make the bonding between BF and the matrix more intact and enhance the interfacial bonding strength between BF and the matrix, thereby improving the mechanical properties and corrosion resistance of the composites [22,129].

1. Mechanical properties

Previous studies have reported that modification of BF with SCAs can significantly improve the mechanical properties of BFRRC. For example, Guo et al. [56] modified the pretreated BF with SCA KH550 to prepare EP resin/BF composites, and further introduced MBF into flame-retardant EP composites (EP/AP750) to prepare BF-reinforced flame-retardant EP composites (EP/AP750/BF-AT). The addition of MBF led to an enhancement in the tensile and impact strengths of EP/BF composites, resulting in effective improvement in the flame-retardant properties of EP/AP750/BF-AT. Lu et al. [19] used SCA KH550 to modify the surface of BF and studied the effects of treatment concentration and soaking time on fiber modification. The results indicated that the addition of coupling-modified fibers could reduce the phase angle and unrecoverable creep compliance of emulsified asphalt evaporation residue; increase the rutting factor and creep recovery rate; and improve the elastic recovery ability and permanent deformation resistance. However, excessive fibers could weaken the ductility of emulsified asphalt at low temperatures. The appropriate content of SCA-MBF was found to be 1.5%. After modification with SCA, the fiber surface became rough, and cohesion between the fiber and the emulsified asphalt base enhanced. To fabricate BF-reinforced cement-based mortars with higher compatibility between reinforcement and matrix, Iorio et al. [21] modified BF using three types of silane aqueous solutions ((i): γ-aminopropyltriethoxysilane (APTES); (ii): γ-aminopropylmethyldiethoxysilane (APDES); and (iii): a mixture of APTES+APDES 50% by weight) and dispersed the MBF in a Portland cement matrix. They reported that better mechanical behavior was achieved when BF modified with a complex mixture of silanes was dispersed in a cement matrix. Furthermore, Iorio et al. [130] also found that three chemical coatings based on amino silane solutions (APTES, APDES, and APTES+APDES) roughened the surface. Thus, they concluded that the higher the amount of triethoxysilane in the composition of the coating solution, the more organic matter was deposited onto the fibers, leading to topographical heterogeneity. This heterogeneity could be responsible for the adhesion between the matrix and the fiber. Arslan et al. [131] investigated the effects of three different SCAs, namely, (3-aminopropyl)triethoxysilane (AP), (3-glycidyloxypropyl)trimethoxysilane (GP), and (3-trimethoxysilyl)propylmethacrylate (MA) on the mechanical properties of the BF-reinforced poly(butylene terephthalate) (PBT) composites. Table 6 summarizes that the addition of SCA led to a significant increase in the tensile strength and elastic modulus of composites, a slight improvement in the flexural strength, and no change in the impact properties. According to the flexural strength and elastic modulus values, the effectiveness of the SCAs can be ranked as follows: GP > AP > MA.

2. Corrosion-resistance properties

When BFRRC is used in corrosive environments such as seawater, its corrosion resistance is critical. Luo et al. [132] used different types of SCAs, i.e., KH550, KH560, and A171, and prepared SCA-modified BF-reinforced polymers. Further, BFRRCs were immersed in a 3.5% NaCl concentration of artificial seawater, 10% H_2_SO_4_, 10% NaOH, and 10% NaCl high-concentration artificial seawater at 20, 40, and 160 °C, respectively. They found that BFRRCs exhibited relatively good resistance to seawater corrosion. The SCA modification enhanced the flexural strength and flexural strength retention by enhancing the interfacial bonding property of BFRRC. The three SCAs modified the corrosive aging performance of the composites in the following order: KH550 > KH560 > A171. Zheng et al. [133] studied the effects of seawater corrosion on the mechanical properties of SCA (KH560) and carboxylated CNT-modified BF-reinforced EP resin (BF/EP) composites by simulating seawater corrosion under different conditions. The synergistic modification of KH560 and CNTs could effectively improve the corrosion resistance of BF/EP composites. Under the same corrosion conditions, the corrosion degree of the MBF composite was found to be lower than that of the untreated BF composite in both distilled water and seawater. This result indicated that the seawater corrosion resistance of the BF-modified/EP was better than that of the BF-untreated/EP. The study is significant for promoting the development of BFRRC and its application in marine environments. Zheng et al. [134] investigated the impact of activating and grafting various SCAs on the mechanical and anti-corrosion properties of chopped BF-reinforced waterborne EP (WEP) coatings. Scanning electron microscopy (SEM) and Fourier-transform infrared (FTIR) spectroscopy analyses confirmed the successful grafting of SCA onto CBF. Mechanical tests, electrochemical impedance spectroscopy (EIS), salt spray aging, and UV-accelerated aging demonstrated significant enhancements in the mechanical properties and corrosion resistance of CBF/WEP coatings.

The above-mentioned research indicates that modification of BF with SCA can significantly improve the interfacial bonding strength between BF and the matrix, thereby enhancing the mechanical properties and corrosion resistance of composites. Most researchers have used several SCAs to modify BF in order to screen out the SCA with the best modification effect. Some scholars have also studied the modification effect of different SCAs on BF by compounding and single doping. Nonetheless, it has been found that the modification effect of SCA compounding is not necessarily better than that of single doping. At present, there is relatively little research on the synergistic effect of SCA co-doped with other materials. Many types of SCAs are available currently, and further research can continue to study the synergistic effects of co-doping between different SCAs.

### 4.2. Rare-Earth-Coupling-Agent-Modified BF

The rare-earth elements belong to a group of elements on the earth that are the rarest and possess unique chemical and physical properties. These include 15 lanthanide elements as well as scandium and yttrium, which are lustrous, silvery-white, soft heavy metals [135,136]. In recent years, rare-earth elements have been found to modify BF, significantly improving its mechanical and durability properties. Owing to the unique 4f valence electron layer structure of rare-earth elements, they possess high chemical activity and can be treated on the surface of fibers through chemical bonding and physical adsorption. This improves the interfacial bonding strength between the fibers and the matrix [137].

In previous studies, researchers have demonstrated the improvement effect of rare-earth coupling agents on the mechanical and durability properties of BFRRC.

1. Mechanical properties

**Figure 17:**
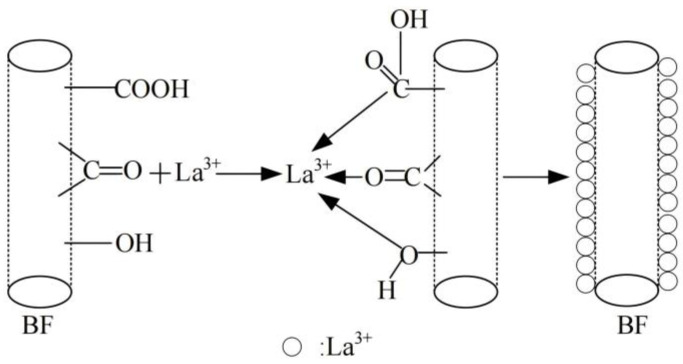
Schematic diagram of La^3+^ attached to the BF surface [137].

Li et al. [138] prepared EP nano-composites with added Ce–Zr–Si oxide for the surface treatment of BF and used MBF to prepare BF/EP resin composites. The results showed that the modified fiber composites could effectively enhance the mechanical properties of EP resin composites. To note, the tensile strength and flexural strength of the hybrid composites reached their peak when 5 wt.% Ce–Zr–Si was added, increasing by 13.8% and 33.67%, respectively. Building upon this research, to further improve the mechanical properties of BFREPC, Li et al. [137] synthesized modified solutions containing different concentrations of lanthanum ions (La^3+^) for modifying the surface of BF. Figure 17 exhibits that after the BFs were soaked in the modification solution, more active groups (C=O, –OH, C–O, etc.) were introduced onto the surfaces of BFs, which effectively enhanced the bond strength between the BFs and the resin matrix. When the La^3+^ content was 0.5 wt.%, the tensile strength, bending strength, and ILSS of the MBF/EP resin composites reached 458.7, 556.7, and 16.77 MPa, respectively. Zhu et al. [139] also used La to enhance BF, modifying BF with lanthanum–ethylenediaminetetraacetic acid (La-EDTA) to improve its mechanical and interfacial properties. The results confirmed the successful introduction of La-EDTA onto the surface of BF as a crystalline lanthanum dihydrate via the reaction between various oxides and the –COOH group of La-EDTA. The average BF monofilament fracture load increased from 124.03 to 163.29 mN after modification. In another related study, Zeng et al. [140] prepared BF with different CeO_2_ contents (0–4 wt.%) by a conventional melting, cooling, and single-hole drawing process and investigated the effect of CeO_2_ doping in BF on the structure, electrical properties, and mechanical properties of the BF. The results showed that for the 2 wt.% weight content of CeO_2_, the glass exhibited the most stable network structure with the largest degree of linkage, resulting in a 75% increase in water resistance, the lowest values of dielectric constant (6.25) and dielectric loss (4.38 × 10^−3^), and a 33% increase in tensile strength.

2. Durability properties

**Figure 18:**
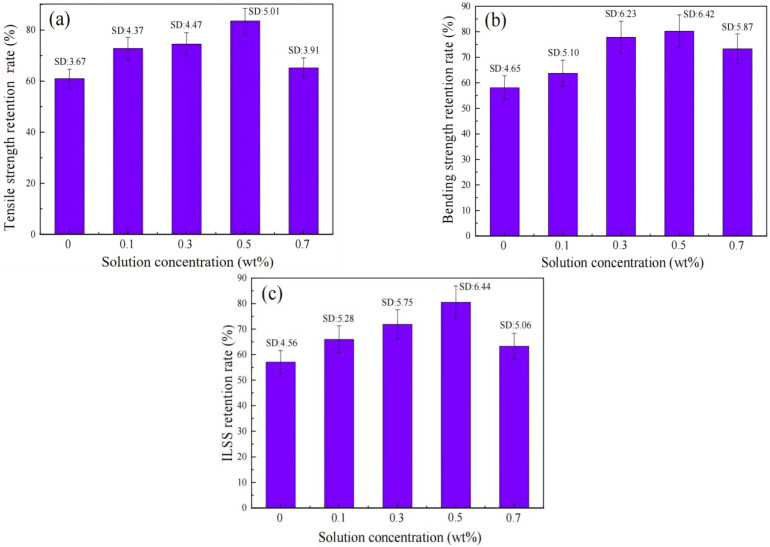
Retention rate of mechanical properties of BF/ERCs after hygrothermal aging: (**a**) retention rate of tensile strength; (**b**) retention rate of bending strength; and (**c**) retention rate of ILSS [141].

Li et al. [141] used CeCl_3_ to modify BF/EP resin composites (BF/ERCs) and studied their resistance to thermal aging. The results indicated an improvement in the hydrothermal-aging resistance of BF/ERCs treated with a rare-earth-modified solution. Figure 18 exhibits that the retention rate of the mechanical properties of BF/ERCs treated with a rare-earth-modified solution containing 0.5 wt.% Ce was up to the peak values. The retention rates of the tensile strength, bending strength, and ILSS of composites exceeded 80% after hygrothermal aging, respectively. Liu et al. [142] added different mass fractions of CeO_2_ to basalt ore, obtained BF-containing CeO_2_ through melting and drawing, and studied its high-temperature resistance. The results indicated that within a certain range, with the increase in the amount of added CeO_2_, Ce^4+^ underwent reduction to Ce^3+^ while promoting the conversion of Fe^2+^ to Fe^3+^ in BF. This strengthened the basalt glass network, increasing the glass transition temperature and improving the high-temperature resistance of BF.

The above-mentioned research results indicate that rare-earth coupling agents exhibit a good non-destructive modification effect on BF. Compared with SCAs, rare-earth coupling agents as modification agents for BF have not been extensively researched to date. The main reasons are as follows:

1. The cost of rare-earth elements is relatively high, coupled with the mining and extraction expenses of rare-earth elements, and their overall cost becomes significantly higher than that of SCAs;
2. The pollution of the ecological environment caused by rare-earth mining and the harm posed to human health caused by long-term use cannot be ignored;
3. The modification process of rare-earth coupling agents for BF is often more complex than that of SCAs.

Therefore, further research on rare-earth coupling agents should focus on their overall use expenses and processing techniques.

SCAs and rare-earth coupling agents used for modifying BF belong to the category of non-destructive modification agents and offer a broad range of application prospects. However, most of the current research on the modification of BF with SCAs and rare-earth coupling agents is still in its infancy, and there is relatively little research on their practical applications. Therefore, the promotion and use of SCAs and rare-earth coupling agents to modify BF remains a major issue to deal with.

## 5. Challenges and Prospects

### 5.1. Challenges

In previous studies, it has been demonstrated that BFRRC presents excellent mechanical, thermodynamic, and durability properties. Therefore, BFRRC has been widely used in many fields such as construction, automotive, aerospace, and power. However, many problems require solving in the research and application areas of BFRRC. A summary of the previous research on BFRRC indicates that BFRRC currently faces the following four main challenges:

1. The interfacial bonding strength of BFRRC still needs to be further improved. To note, the interfacial bonding strength directly affects the mechanical properties and application prospects of BFRRC. In previous studies, fiber modification was often used to improve the interfacial bonding strength, and matrix modification has also been reported. However, some studies have found that BFRRC reinforced via fiber modification or matrix modification still undergoes the phenomenon of disadhesion between the fibers and the matrix during the material performance. This can affect its applications; in particular, when BFRRC is exposed for a longer duration to the external environment or erosion environments [113];
2. Inadequate research on the properties of BFRRC limits its practical application. To date, extensive research efforts have been devoted to studies on the mechanical and thermodynamic properties of BFRRC; nonetheless, relatively few studies have been carried out on the durability of BFRRC. Moreover, some durability studies have found that the effect of BFRRC is weakened or lost [77,85,113]. In addition, most of the studies on the durability of BFRRC have been simulated in the laboratory, and the experimental data may be quite different from the results obtained after actual application, which are not convincing enough. Further, only a few studies are available on the durability of BFRRC used in practical scenarios;
3. The knowledge about the economic aspects of the new BF-surface-modification method is insufficient. The traditional destructive surface-modification technique of BF includes acid–base treatment and plasma modification. However, acid–base modification inevitably causes certain losses to the silicate skeleton of BF, and the improvement in the interfacial properties of BFRRC is limited. Therefore, progressively more attention has been paid to the non-destructive surface-modification technique. However, agents used in non-destructive surface modification, such as SCAs and rare-earth coupling agents, are often expensive and complex, thus, it becomes difficult to achieve mass production in industrial applications [143];
4. Recycling of BFRRC is another problem that is inevitably encountered. With the continuous promotion and application of BFRRC, the recycling of BFRRC at the end of its service life will be a challenging task.

### 5.2. Prospects

In response to the four above-mentioned challenges, future research on BFRRC can be carried out from the following aspects:

1. First, it is required to improve the interfacial bonding strength of BFRRC—in particular, to ensure its safe and efficient long-term exposure to the external environment and erosion environments. In the follow-up study, research attempts should be made to improve the interfacial bonding strength of BFRRC by combining BF surface modification and matrix modification techniques or adding adhesive materials with good compatibility with BF and the resin matrix;
2. Research should be conducted on the durability of BFRRC as this directly determines its service life and cost. Resin materials are usually degradable, as indicated by biological and abiotic factors [85]. Therefore, improvement in the durability of BFRRC is significantly important. In the follow-up study, attempts should be made to add filler materials to improve the compactness of BFRRC and reduce the passage of corrosive media such as seawater into the interior of BFRRC. This approach can improve the durability of BFRRC in corrosive environments such as seawater. Moreover, materials such as engineering ceramics can also be added to improve the thermal stability of BFRRC, which can eventually improve the durability of BFRRC in high-temperature environments;
3. Third, it is also imperative to study the methods for reducing the cost of preparation of BFRRC. To note, the cost of material production is the key factor that restricts its widespread use. The non-destructive surface-modification technology of BF developed in recent years—in particular, the method of preparing BF modified with rare-earth coupling agents—involves a significantly higher production cost than the previously reported destructive surface-modification technique. Unfortunately, the high production cost directly limits the popularization and application of non-destructive surface-modification technology. In the future, the cost can be reduced through the synergistic effects of the composite used with other low-cost fiber-modified materials, among which nanomaterials offer great potential as the most suitable candidates. Moreover, simplification of the manufacturing process of BFRRC can also effectively reduce the costs;
4. Further research should be conducted on the recycling of BFRRC. In the next few decades, the BFRRCs in service will be eliminated one after another due to factors such as the end of service life or replacement requirements, and the recycling of many eliminated BFRRCs face serious challenges. Therefore, the recycling issues of BFRRC should be considered in its manufacturing stage, including the classification and recycling of BF- and resin-based materials.

In addition to the above prospects, BFRRC is also involved in the development of high-performance composite materials, such as modification and reinforcement through the integration of nanomaterials. BFRRC’s green manufacturing processes, the development of sustainable production models that reduce energy consumption and carbon emissions, as well as the intelligent applications of BFRRC—such as integrated innovations in sensors and smart structures—are areas for future research. In summary, the research prospects of BFRRC are broad and worthy of in-depth exploration by researchers.

## 6. Conclusions

The preparation technology, properties, and application status of BF and resin matrix composites are reviewed herein. This review further emphasizes the influence of non-destructive surface-modification methods on the properties of BFs. After reviewing and discussing the collected literature, the following conclusions can be drawn:

1. Compared with traditional GF, BF offers significant economic and environmental benefits. BF-reinforced resin matrix composites (BFRRCs) prepared with BF have stable properties. In the future, BF can be used to partially or completely replace GF as the reinforcement material of FRRCs;
2. The mechanical properties, thermodynamic properties, and durability of BF-enhanced resin-based composites can generally be improved by several-tens to several-thousand percent. Nonetheless, the interfacial bonding strength, durability, and overall properties of BFRRC still need to be further improved;
3. The mechanical properties of BFRRCs can be improved by BF modification, resin matrix modification, the addition of a reinforcement agent, and BF mixed with other fibers. In the follow-up research, the appropriate BFRRC enhancement method can be selected according to the performance and cost requirements;
4. Non-destructive surface-modification techniques such as silane coupling agents and rare-earth coupling agents can effectively improve the overall properties of BFRRCs. However, the cost of non-destructive surface-modification technology is too high, which leads to fewer practical applications. Thus, undeniably, a lot more systematic exploration is further demanded for cost reduction and practical application effects;
5. BFRRC is a light-weight, high-strength, energy-saving, and environmentally sustainable material with excellent comprehensive properties. It can be widely used in the fields of construction, aerospace, automobiles, and power;
6. In the future, BFRRC research can be advanced in the areas of developing high-performance composite materials, the green manufacturing process of BFRRC, and intelligent applications of BFRRC. In addition, researchers should strengthen the integration of industry, academia, and research to promote technology transformation.

## Figures and Tables

**Table 1:** Thermal properties of BFs and GFs [33].

Thermal Properties	BFs	GFs
Maximum application temperature (K)	1255	923
Sustained operating temperature (K)	1093	753
Minimum operating temperature (K)	15	210
Thermal conductivity (W·(m·K)^−1^)	0.031–0.038	0.034–0.04
Melting temperature (K)	1720	1390
Thermal expansion coefficient (K^−1^)	8.0 × 10^−6^	5.4 × 10^−6^

**Table 2:** Electrical properties of BFs and E-GFs [34,35].

Property	BFs	E-GFs
Volume resistivity (Ω·m)	1 × 10^12^	1 × 10^11^
Loss angle tangent frequency (1 MHz)	0.005	0.0047
Relative dielectric constant (1 MHz)	2.2	2.3

**Table 3:** Chemical properties of BFs and GFs [36,37].

Property	BFs	E-GFs	S2-GF
Quality loss rate after boiling for 3 h (wt.%)	0.4	6.2	5
Quality loss rate of NaOH after boiling for 3 h (wt.%)	4.3	6	5
Quality loss rate of HCl after boiling for 3 h (wt.%)	8.1	38.9	15.7

**Table 4:** Effect of adding BF on the mechanical properties of the resin matrix.

Type of Resin	The Mechanical Properties of BFRRC				Ref.
	Tensile Strength	Flexural Strength	Bending Strength	Impact Strength	
EP resin	-	-	-	increased by 339.3%	[42]
UPR	increased by 2626%	increased by 804%	-	-	[43]
PE resin	increased by 87.8%	increased by 132.6%	-	-	[44]
PE resin	increased by 59.7%	increased by 175%	-	-	[45]
PP resin	increased by 133%	-	-	-	[24]
PA resin	-	-	increased by 70%	-	[46]

**Table 5:** Effect of surface modification of BF on mechanical properties of BFRRC.

BF-Surface-Modification Material	Modification Method	Matrix	Improved Mechanical Properties	Ref.
ZnO	Electroless deposition	EP resin	Apparent interfacial shear strength increased by 42%.	[55]
Graphene oxide	Electrophoretic deposition	EP resin	Tensile strength increased by 13.2% and coefficient of friction reduced by 18.1%.	[20]
Tetravinylsilane	Plasma polymerization technique	UPR	Interlaminar shear strength above 180%.	[63]
Methacrylic acid and KH-570	Surface coating	Phenolic epoxy resin	Compressive strength and flexural strength of MBF cement stone increased by 15.9% and 13.5%, respectively.	[73]
Cellulose nanocrystals	-	PP resin	Mechanical strength increased by 64.31%.	[92]
Graphene	Polyurethane sizing agent coating	PA resin	Tensile strength and flexural strength increased by 18.2% and 34%, respectively.	[105]
3-Aminopropyltriethoxysilane	Plasma polymerization technique	PA resin	Interfacial shear strength and tensile strength increased by 50.3% and 32.5%, respectively.	[106]

**Table 6:** Effect of SACs on the mechanical properties of composites [131].

Sample Code	Tensile Properties			Flexural Properties		Impact Properties
	Tensile Strength (MPa)	Percentage Strain (%)	Young’s Modulus (MPa)	Flexural Strength (MPa)	Flexural Modulus (MPa)	Impact Strength (kJ·m^−2^)
PBT	48.9 ± 0.54	22.2 ± 0.53	1900 ± 200	48.9 ± 0.54	2400 ± 50	29.9 ± 1.40
PBT/BF	47.6 ± 0.89	3.1 ± 0.23	3600 ± 100	47.6 ± 0.89	4400 ± 20	20.0 ± 0.31
PBT/AP-BF	63.4 ± 1.75	6.2 ± 0.22	3700 ± 200	63.4 ± 1.75	4200 ± 70	24.0 ± 1.95
PBT/GP-BF	62.0 ± 0.92	4.3 ± 0.40	3600 ± 200	62.0 ± 0.92	4500 ± 210	20.5 ± 0.31
PBT/MA-BF	61.9 ± 1.07	5.3 ± 0.05	3600 ± 200	61.9 ± 1.07	4200 ± 220	21.7 ± 0.94

## Data Availability

No new data were created or analyzed in this study. Data sharing is not applicable to this article.
